# Central hypothyroidism in a pediatric case of primary acute monoblastic leukemia with central nervous system infiltration: A case report

**DOI:** 10.1097/MD.0000000000007329

**Published:** 2017-06-30

**Authors:** Yuya Sato, Satomi Koyama, Shigeko Kuwashima, Masaya Kato, Mayuko Okuya, Keitaro Fukushima, Hidemitsu Kurosawa, Osamu Arisaka

**Affiliations:** a Department of Pediatrics; b Department of Radiology, Dokkyo Medical University School of Medicine, Mibu, Tochigi, Japan.

**Keywords:** CNS infiltration, leukemia, MLL rearrangement with AF9, thyroid-stimulating hormone, thyroxine

## Abstract

**Rationale:**

Central nervous system (CNS) leukemia is a frequent diagnosis in pediatric acute myeloblastic leukemia (AML) and includes neural symptoms. However, CNS leukemia is rarely associated with central hypsothyroidism.

**Patient concerns and diagnoses:**

A 2-year-old female with AML with MLL rearrangement presented with CNS infiltration. Laboratory tests suggested the presence of central hypothyroidism (thyroid-stimulating hormone [TSH]: 0.48 mIU/ml, normal range 0.7–6.4 mIU/ml; serum free thyroxine [FT4]: 0.62 ng/dl, normal range 0.8–2.2 ng/dl; free triiodothyronine: 1.57 pg/ml, normal range 2.7–5.6 pg/ml). Magnetic resonance imaging detected no lesions in the hypothalamus, pituitary, or thyroid.

**Interventions and outcomes:**

Levothyroxine (2.5 mg/kg/day) was administered together with chemotherapy and intrathecal injection of methotrexate, cytarabine, and hydrocortisone into the cerebrospinal fluid. The FT4 concentration increased after levothyroxine treatment, but later decreased after relapse of CNS leukemia. The TSH concentrations remained low. After remission of CNS leukemia, the TSH and FT4 concentrations quickly recovered to their normal ranges.

**Lessons:**

We believe that the CNS leukemia directly affected TSH and thyroid hormone secretion in our patient.

## 1 Introduction

Extramedullary leukemia is a common finding in patients with acute myeloblastic leukemia (AML).^[1]^ Central nervous system (CNS) leukemia is a frequent diagnosis in pediatric AML.^[2,3]^ Although CNS leukemia does not apparently affect overall survival,^[2,3]^ it is a risk factor for CNS relapse.^[4]^ In fact, CNS leukemia is an important determinant of the choice of treatment.^[4,5]^ Patients with CNS leukemia receive reinforced treatment, including radiotherapy for CNS, intrathecal injection of methotrexate, or stem cell transplantation. These treatments have improved the recovery of patients with CNS leukemia. CNS leukemia often causes neural symptoms such as vomiting, nausea, and paralysis.

Pediatric leukemia survivors, especially those who developed CNS leukemia, are at increased risk of developing hypothalamic and pituitary disorders.^[6,7]^ Hypothyroidism is one of the main complications of chemotherapy or stem cell transplantation in leukemia survivors. Many studies have focused on treatment-related hypothalamic and pituitary disorders in pediatric leukemia patients.^[6–11]^ However, hypothyroidism is seldom directly caused by CNS leukemia. In this paper, we report central hypothyroidism in a pediatric case of primary AML with CNS infiltration.

## 2 Case report

**Figure 1:**
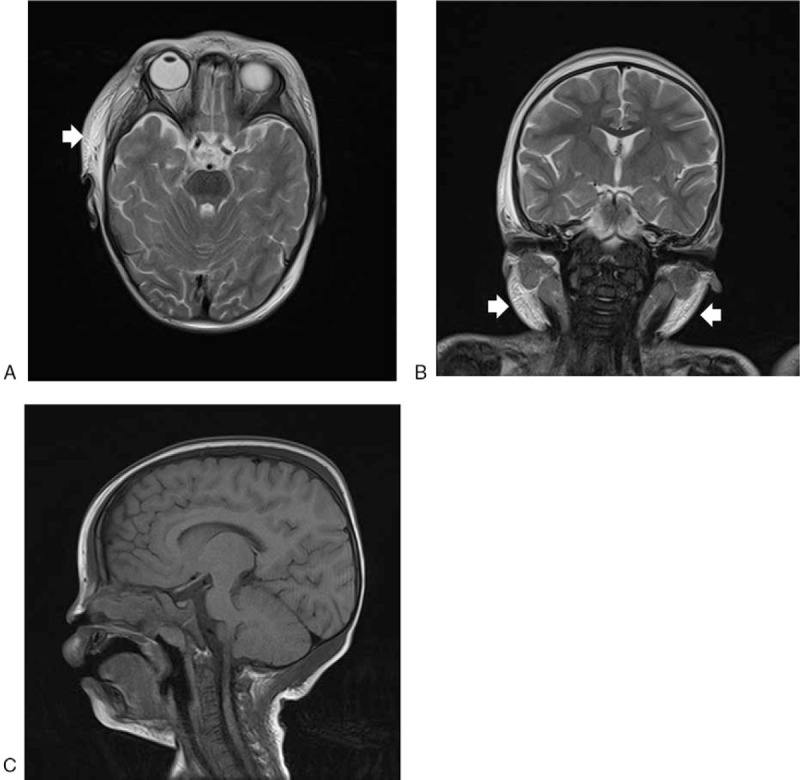
Magnetic resonance imaging findings. (A) Axial T2-weighted image; (B) coronal T2-weighted image; (C) sagittal T1-weighted image. Myeloid sarcomas are visible as hyperdense ill-defined subcutaneous tumors on the T2-weighted images (arrows). No lesions are evident in the brain. Specifically, there are no apparent abnormalities in the pituitary gland or hypothalamus.

A 2-year-old girl was admitted to our hospital with a diagnosis of acute monoblastic leukemia with MLL rearrangement with AF9 (MLL/AF9). The patient had no problems with her general health, development, or growth before the onset of leukemia. Leukemic blasts (11 cells/μL) with MLL/AF9 had infiltrated the cerebrospinal fluid (CSF), but there were no neural symptoms. Magnetic resonance imaging (MRI) revealed myeloid sarcomas subcutaneously in the temporal region and infragnathia, but no lesions were found in the brain, including the pituitary gland and hypothalamus (Fig. 1). The thyroid was not swollen. The laboratory tests were suggestive of central hypothyroidism (thyroid-stimulating hormone [TSH]: 0.48 μIU/mL, normal range 0.7–6.4 μIU/mL; serum free thyroxine [FT4]: 0.62 ng/dl, normal range 0.8–2.2 ng/dl; free triiodothyronine: 1.57 pg/mL, normal range 2.7–5.6 pg/mL).

The patient was given chemotherapy, comprising intrathecal injection of methotrexate (10 mg/dose), cytarabine (25 mg/dose), and hydrocortisone (20 mg/dose). Chemotherapy led to the immediate disappearance of leukemic blasts from the CSF and bone marrow. However, the leukemic blasts increased (283 cells/μL) and MLL/AF9 was detectable in CSF at 4 weeks after starting chemotherapy. Therefore, the patient was diagnosed with relapsed CNS leukemia. Three times of intrathecal injections (12 mg of methotrexate, 25 mg of cytarabine, and 20 mg of hydrocortisone), which were performed every week, led to the disappearance of leukemic blasts at week 6 and MLL/AF9 was not detectable in the CSF.

Even though the patient had no symptoms associated with hypothyroidism, levothyroxine was started at the minimum dose (2.5 μg/kg/day) on day 1. The FT4 concentration increased after 4 weeks of levothyroxine administration. Levothyroxine was continued, but FT4 decreased to 0.86 ng/dL at week 6, when the relapse of CNS leukemia was detected. TSH remained at low concentrations for 6 weeks after starting chemotherapy, and the TSH and FT4 concentrations recovered immediately to their normal ranges once remission of CNS leukemia was achieved in week 7. The administration of levothyroxine was suspended at week 18 because the TSH and FT4 concentrations had remained within their normal ranges.

## 3 Discussion

Very few patients with hypothyroidism and primary leukemia have been reported to date. Foresti et al^[12]^ reported a patient with acute B cell lymphoblastic leukemia who developed primary hypothyroidism. The authors reported that direct infiltration of leukemic blasts to the thyroid induced hypothyroidism in their patient, who had elevated TSH concentrations and low FT4 concentrations.^[12]^ In our patient, the thyroid was not swollen and the TSH concentration was not elevated at diagnosis. These findings suggest that the thyroid dysfunction in our patient was not due to the infiltration of leukemic blasts into the thyroid.

Lei et al^[13]^ reported that high neonatal TSH concentrations were associated with increased risk of pediatric leukemia. Verbeek et al and Tanouchi et al^[14,15]^ reported the development of leukemia in patients with congenital hypothyroidism. However, TSH did not affect the occurrence of leukemia because the bone screening tests were negative and the TSH concentrations were low in our patient at the time of admission.

In our patient, hypothyroidism appeared to be due to the CNS infiltration of leukemic blasts, after which the TSH and FT4 concentrations changed markedly. The TSH and FT4 concentrations returned to within their normal ranges upon remission of CNS leukemia. However, MRI did not reveal any leukemic lesions in the pituitary gland or hypothalamus. Ranta et al^[16]^ reported that CNS lesions were not detected by MRI in 15/21 patients with acute lymphoblastic leukemia and CNS involvement and symptoms. Because of the low number of leukemic blasts, they were not detectable by MRI. The presence of blasts impaired the production of TSH in the anterior lobe of the hypophysis, and hence impaired the secretion of thyroid hormones.

Our findings in this patient indicate that central hypothyroidism may occur in patients with CNS leukemia. We think that the leukemic blasts had infiltrated the pituitary gland and hypothalamus. Although these blasts could not be detected by MRI, they could affect the secretion of TSH and thyroid hormone.

## 4 Conclusions

CNS leukemia may directly affect TSH and thyroid hormone secretion, and induce central hypothyroidism in some patients with acute monoblastic leukemia.
